# Automated tick classification using deep learning and its associated challenges in citizen science

**DOI:** 10.1038/s41598-025-10265-x

**Published:** 2025-07-10

**Authors:** Anna Omazic, Giulio Grandi, Stefan Widgren, Joacim Rocklöv, Jonas Wallin, Jan C. Semenza, Najmeh Abiri

**Affiliations:** 1 Department of Chemistry, Environment and Feed Hygiene, Swedish Veterinary Agency (SVA), 751 89 Uppsala, Sweden; 2 Department of Animal Biosciences, Swedish University of Agricultural Sciences (SLU), 750 07 Uppsala, Sweden; 3 Department of Epidemiology, Surveillance and Risk Assessment, Swedish Veterinary Agency (SVA), 751 89 Uppsala, Sweden; 4 Department of Public Health and Clinical Medicine, Section of Sustainable Health, Umeå University, 901 87 Umeå, Sweden; 5 Heidelberg Institute of Global Health, University of Heidelberg, Heidelberg, Germany; 6 Department of Statistics, Lund University, 221 00 Lund, Sweden; 7 School of Information Technology, Halmstad University, 301 18 Halmstad, Sweden

**Keywords:** Computer science, Infection, Ecological epidemiology

## Abstract

Lyme borreliosis and tick-borne encephalitis significantly impact public health in Europe, transmitted primarily by endemic tick species. The recent introduction of exotic tick species into northern Europe via migratory birds, imported animals, and travelers highlights the urgent need for rapid detection and accurate species identification. To address this, the Swedish Veterinary Agency launched a citizen science initiative, resulting in the submission of over 15,000 tick images spanning seven species. We developed, trained, and evaluated deep learning models incorporating image analysis, object detection, and transfer learning to support automated tick classification. The EfficientNetV2M model achieved a macro recall of 0.60 and a Matthews Correlation Coefficient (MCC) of 0.55 on out-of-distribution, citizen-submitted data. These results demonstrate the feasibility of integrating AI with citizen science for large-scale tick monitoring while also highlighting challenges related to class imbalance, species similarity, and morphological variability. Rather than robust species-level classification, our framework serves as a proof of concept for infrastructure that supports scalable and adaptive tick surveillance. This work lays the groundwork for future AI-driven systems in One Health contexts, extendable to other arthropod vectors and emerging public health threats.

## Introduction

Tick-borne diseases pose a growing animal and public health threat, particularly in Europe, where the spread of zoonotic diseases transmitted by the Ixodidae family of ticks has been exacerbated by climatic changes. Rising temperatures and changing environmental conditions have contributed to improved tick survival and increased tick abundance, allowing for a broader geographical spread of tick-borne pathogens ^1–3^. The most important vector-borne diseases in Europe, Lyme borreliosis and tick-borne encephalitis (TBE), are transmitted by the primary tick vector, *Ixodes ricinus*. Both Lyme disease and TBE have increased in incidence over the last decade, with possible explanations, including the geographic expansion of the ticks involved in transmission and improved diagnostic awareness, reducing historical under-reporting ^4^. Ticks are also responsible for a wide range of transmission of other tick-borne pathogens that can cause disease in animals and/or humans. Of particular concern is the importation of exotic ticks on migratory birds that could transmit diseases such as Crimean Congo Hemorrhagic Fever in areas where the disease has not occurred yet^5^. These evolving circumstances and this increasing risk from tick-borne diseases pose new challenges for public health practice that need to be tackled with novel strategies and innovative solutions.

Traditionally, entomologists have collected and compiled tick distribution data for public health purposes. One such example is VectorNet, which is a network of medical and veterinary experts and organizations that maintain a database on the presence and distribution of vectors and pathogens in vectors across Europe and the Mediterranean basin ^6^. It supports the collection of data on vectors and pathogens in vectors related to both animal and human health with the aim to advance preparedness and response for vector-borne diseases. These entomological data are integral to the One Health approach that integrates animal, human, and environmental health surveillance, which is a more comprehensive approach to tackling the emergence, transmission, and dispersion of infectious diseases. Moreover, this One Health strategy can be operationalized across several spatial domains (local, national, regional, and global) and temporal scales (tracking historical changes, short-term predictions, and long-term projections) to elucidate the impact of climate change on emerging infectious diseases. However, the collection of wide-ranging entomological data as a crucial pillar of One Health surveillance has proven to be resource, time, and labor-consuming and nonfeasible for larger-scale surveillance, such as for a country. To address this gap, citizen science initiatives have emerged as a powerful and cost-effective tool for collecting extensive and geographically diverse data on a wider geographical scale and for longer periods.

In Sweden, a recent citizen science project has engaged the general public to contribute to tick surveillance ^7^. In just one year, participants submitted around 18,000 images of ticks, along with metadata about where and when ticks were found and information about the host (e.g., domestic or wild animals or humans). Experts at the Swedish Veterinary Agency (SVA), Uppsala, Sweden, verified and labeled these contributions, greatly enhancing the existing data on both endemic tick species and exotic ones. However, the sheer volume of data renders the processing of submissions impractical. Thus, automating the process of tick species identification offers the prospect of advancing both efficiency and scalability. Automating tick classification using image-based machine learning techniques offers a promising solution. Advances in image classification, particularly through state-of-the-art models such as convolutional neural networks (CNNs) and transformers, have demonstrated remarkable success across various biological applications, including the classification of insects. Recent research has explored the application of machine learning to insect identification, highlighting the potential for these models to aid in species classification. For instance, neural networks have been used effectively to identify and classify ticks and other arthropods in large datasets, with promising results ^8–12^. These approaches have demonstrated the feasibility of using deep learning models to process complex biological images, making them ideal candidates for automated tick classification systems.

A critical aspect of deploying machine learning models for tick classification is ensuring the model’s ability to generalize to new, unseen data. In-distribution (ID) testing evaluates model performance on data with the same distribution as the training set. However, real-world applications often encounter out-of-distribution (OOD) data, where the characteristics of the input samples differ from the training data in terms of species, geographical locations, or image quality. OOD testing is essential because it assesses how well a model can generalize beyond the specific conditions on which it was trained. It makes it crucial for practical, real-world deployments of AI systems in fields like tick-borne disease monitoring. Proper evaluation of both ID and OOD performance is key to ensuring robust and reliable predictions, especially when handling diverse citizen-submitted data sources ^13,14^. As tick classification models are expected to handle data from various environmental conditions, hosts, and camera qualities, incorporating OOD testing helps assess the model’s readiness for practical use in variable settings.

Besides morphological differences that exist between different tick species, even within the same tick species, the morphological identification can be challenging ^15–17^. This is because ticks have a complex life cycle, including different developmental stages (larva, nymph, male, and female) varying in size and shape. In addition, the same developmental stage can greatly differ before and after engorgement. Automating tick classification using image-based machine learning techniques offers a promising solution.

Here, we build on these novel methods to citizen science data and implement advanced image classification techniques for the automated detection and classification of tick species. By leveraging the latest developments in AI, the objective was to create a robust and scalable solution that can handle large volumes of data and improve accuracy in classifying visually similar species from citizen science. Moreover, integrating explainable AI (XAI) methods, such as the Randomized Input Sampling for Explanation (RISE) ^18^, will help ensure transparency and trust in the model’s predictions when applied to citizen science data for tick classification. Technical challenges such as data imbalance and species similarity had to be overcome in order to bring these machine learning techniques to fruition. By building on previous research and integrating state-of-the-art techniques, this work contributes to the development of automated tools that can assist researchers and the public in monitoring and responding to the evolving threat of tick-borne diseases in Europe and beyond. .

## Materials and methods

### Tick species

**Fig. 1:**
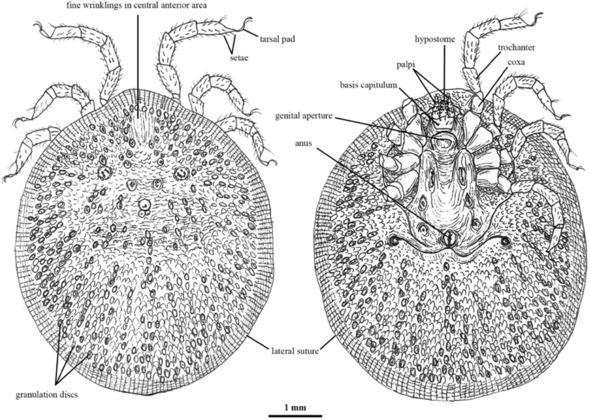
Image of *C. vespertilionis* taken from article ^22^.

In this chapter, we begin by describing the morphological features of tick species that are relevant to Sweden and are under investigation by SVA. These species are either endemic or have been identified in Sweden (at the regional level or in the whole country) or nearby regions (i.e., Northern European countries), indicating a potential risk of their spread within the country. Accurate identification of these species is essential for assessing and mitigating associated animal and public health risks. The general tick glossary is extracted from the book “Ticks”  ^16^ and European Scientific Counsel Companion Animal Parasites (ESCCAP) ^15^. We want to clarify that we are focusing on a select set of prominent morphological features for each species, particularly those that are more visually distinct and likely to be more easily detected by a deep learning model. The tick species included are as follows:

*Carios (C.) vespertilionis* (endemic): Commonly referred to as the bat tick, *C. vespertilionis* is a soft (argasid) tick and primarily a bat parasite. Its host-specific behavior makes it relevant in the context of pathogen transmission among bat populations, even if its role in the transmission of zoonotic pathogens to humans cannot be excluded ^19^. The morphological analysis of *C. vespertilionis* reveals distinct body structures (Figure 1) characterized by wrinkling patterns in the anterior area and granulation discs, which indicate specialized adaptations for bat host attachment. These features facilitate its classification and differentiation from other species ^20,21^.

**Fig. 2:**
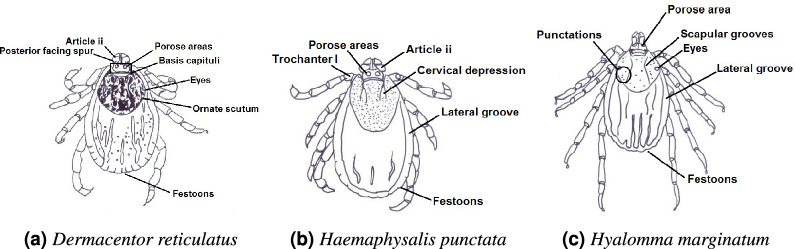
This image is provided for research and educational purposes only. The image is protected by copyright and may not be reproduced, distributed, or used without explicit permission from ESCCAP UK and Ireland.

*Dermacentor (D.) spp.*(exotic, sporadically reported in Sweden): The *Dermacentor* genus has over 35 species, with both *D. reticulatus* and its closely related sibling, *Dermacentor marginatus*, found in Europe ^16^. *Dermacentor reticulatus* (Figure 2a) known as the ornate cow tick or marsh tick, *D. reticulatus* is distributed across Europe and Asia. It infests a broad range of hosts, including humans, and is a recognized vector for diseases such as babesiosis and rickettsiosis. The scutum’s characteristic patterns, coupled with the festoons along the body’s periphery, serve as primary visual markers for identifying this genus ^23,24^. In June 2024, the first case of *Dermacentor marginatus* was reported to the SVA ^25^. Due to the rarity of this species and its close similarity to *Dermacentor reticulatus*, we have chosen to focus our data collection efforts solely on *Dermacentor reticulatus*.

*Haemaphysalis (H.)punctata* (endemic, regional occurrence in Sweden): Widely distributed across Europe and Asia, *H. punctata* (Figure 2b) demonstrates high host adaptability, feeding on birds, mammals, and occasionally humans. This species plays a role in the transmission of multiple pathogens. Key morphological features include the absence of eyes, a smooth, rounded body, prominent festoons, and a lateral groove, which are critical for species identification ^26,27^.

*Hyalomma spp.* includes several species of ticks, two of which, *Hyalomma marginatum* and *Hyalomma rufipes*, have been sporadically reported in Northern Europe ^28,29^. Both species are known vectors of the Crimean-Congo hemorrhagic fever virus. However, due to limited data availability, we focus only on *H. marginatum* in this study. *H. marginatum* (Figure 2c) is commonly found in southern Europe, North Africa, and parts of Asia. Its adult stages primarily feed on large mammals like horses and livestock. Morphological analysis reveals distinct features, including an elongated body, long mouthparts, prominent scapular grooves, and legs adapted for rapid movement.

**Fig. 3:**
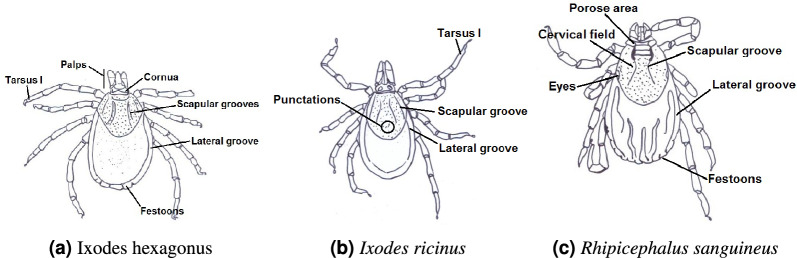
These images are provided for research and educational purposes only. They are protected by copyright and may not be reproduced, distributed, or used without explicit permission from ESCCAP UK and Ireland.

Ixodes (*I*.) hexagonus (endemic): Commonly known as the hedgehog tick, *I. hexagonus* parasitizes hedgehogs, dogs, and cats and is noted for its potential to spread Lyme disease. Morphological examination highlights a small, compact body with fine cervical grooves, essential traits for distinguishing it from other species (Figure 3a). A key visual indicator of the species is the lighter or whitish coloration, especially in empty adult females, which further aids in identification ^16,30,31^.

*Ixodes (I.) persulcatus* (endemic, regional occurrence in Sweden): *I. persulcatus* is a significant vector for diseases such as Lyme borreliosis and tick-borne encephalitis, predominantly found in northeastern Europe and northern parts of Asia. The species exhibits distinctive lateral grooves and a dark, compact scutum. However, it closely resembles *Ixodes ricinus*, making visual identification with the naked eye challenging. It can be challenging even for an expert taxonomist to differentiate this species from *I. ricinus*, since some of the most useful structures (e.g., the marginal groove in the female tick) can disappear as soon as the tick becomes partially engorged. Both morphological and molecular identification is usually performed to confirm the identification of this species  ^16,30,32^. In our study, we omitted this class since there was no evidence to prove an uploaded image in the web application is from this species.

**Fig. 4:**
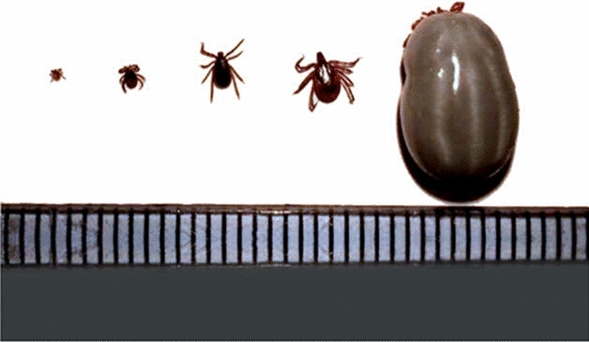
From left to right, *Ixodes ricinus*; larva, nymph, unfed adult male, unfed adult female, fully engorged adult female (scale mm). This image is provided for research and educational purposes only. The image is protected by copyright and may not be reproduced, distributed, or used without explicit permission from ESCCAP UK and Ireland.

*Ixodes (I.) ricinus* (endemic): Commonly referred to as the sheep tick or castor bean tick, *I. ricinus* is the most prevalent tick species in Sweden and a major vector of Lyme disease in Europe. It also transmits diseases such as anaplasmosis and babesiosis. Morphological analysis reveals a round body with no eyes and distinct punctations on the scutum (Figure 3b). However, these features may not be sufficient for distinguishing it from closely related species like *I. persulcatus* ^16,30^. We also include images of different life stages (Figure 4), as this species is the most frequently reported tick in Sweden, warranting a more comprehensive description.

*Rhipicephalus (R.) sanguineus species group* (exotic, sporadic reports in Sweden): Known as the brown dog tick, *R. sanguineus species group* has a global distribution and is commonly associated with dogs. It is a vector for several canine diseases, including ehrlichiosis and babesiosis, and can also transmit pathogens like *Rickettsiae* to humans, potentially causing rickettsial diseases such as Mediterranean spotted fever. The species is characterized by a long, narrow body with festoons along the edge, as well as unique scutal patterns that aid in its identification (Figure 3c). The brown dog tick can complete its lifecycle indoors, making it particularly adept at infesting homes and kennels ^33,34^. Given recent taxonomic developments, we refer to this as the *Rhipicephalus sanguineus* species group rather than a single species ^35^.

All species have been documented for the development of our classification system, with specific anatomical features presented in corresponding images to enhance species identification and facilitate further analysis.

The ixodid ticks life cycle is hemimetabolous and comprises four stages: egg, larva, nymph, and adult. Each stage requires a blood meal to progress, with ticks undergoing metamorphosis at each transition ^36^. Here, we briefly describe tick development’s primary stages and appearance. Since there are many variations in the life cycle patterns depending on the tick species, we mainly refer to the development of *Ixodes* spp. ticks in the following section.

Egg: Ticks begin their life cycle as eggs, which are laid in large quantities by adult females, usually after engorgement is completed and in variable amounts depending on the tick species. After hatching, the larvae develop as the first mobile stage.

Larva (six-legged stage): In the larval stage, ticks have six legs, represent the smallest developmental form, and lack spiracular openings (stigma). They typically feed on small mammals or birds, which leads the larvae to transition into the nymph stage.

Nymph (eight-legged stage): Ticks enter the nymphal stage after molting with two additional legs and the appearance of spiracular openings. Nymphs are larger than larvae but smaller than adults. In this stage, ticks frequently feed on larger animals and can transmit pathogens, although their forms remain less developed than those of adult ticks.

Adult: The adult stage signifies sexual maturity, with noticeable distinctions between males and females, including the appearance of the genital opening on the ventral side of the body. Female ticks require a blood meal for reproduction, becoming engorged after feeding, which precedes egg production. In contrast, males typically do not feed extensively; instead, they remain on the host primarily to locate and mate with females, often attaching to the same host for this purpose.

Morphological Variation Across Developmental Stages: The morphology of ticks varies significantly throughout their life cycle, complicating identification based on images alone, particularly for AI systems. Nymphs, for instance, are small and less morphologically distinct, making them hard to differentiate from larvae or adults in images. Additionally, engorgement after feeding can obscure key features, especially in larvae, nymphs, and females, which challenges AI-based classification. However, in traditional morphological identification methods, these variations are less problematic when directly examining specimens. This highlights the difficulty in image-based species identification.

### Data sources

In this part, we describe the data sources, their distribution, and the part of the process in which they were used.

#### Global biodiversity information facility (GBIF)

To access images of invasive tick species, we relied predominantly on online sources, particularly GBIF. We were able to obtain images for all seven species from this source^37–43^. However, we encountered several challenges while preprocessing the data, such as mislabeled specimens and poor-quality images. These issues necessitated a manual review of each image to verify both its quality and labeling. In cases where we had doubts, the images were further validated by experts at SVA. The distribution of this dataset is detailed in Table 1. This data was utilized for model training, validation, and in-distribution testing.

#### Tick photography (TickExpand)

With the support of parasitologists from SVA, we were able to photograph 18 frozen tick specimens from 5 different species (*Dermacentor reticulatus*, *Haemaphysalis punctata*, *Hyalomma marginatum*, *Ixodes hexagonus*, *Rhipicephalus sanguineus*) in a laboratory setting. The photography has been done using three different smartphones and an external lens. We used mobile phone cameras to reflect the data we would obtain through Citizen Science. Several photos were taken from each sample at different angles and lighting, aiming to reproduce the lighting effects and position of a Citizen Science sample. This data has only been used to train the model to avoid any data leakage. The data frequency distribution is shown in table 1.

**Fig. 5:**
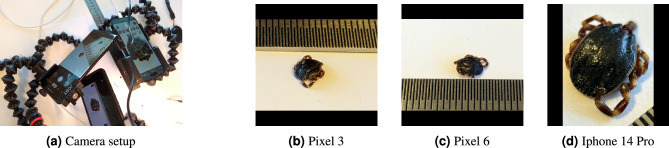
(**a**) Camera setup. (**b-d**) Three shots from one sample, *Hyalomma marginatum*, with different cameras.

We used three smartphone cameras to capture images for the ExpandTick dataset to include a wide variety of image qualities and perspectives. The first device was a Google Pixel 3 smartphone with a focal length of 4.44 mm, providing standard imaging capabilities for our dataset. The second device was a Google Pixel 6 Pro, with a focal length of 6.81 mm, offering higher-resolution images and improved imaging performance. Lastly, we used an iPhone 14 Pro with a focal length of 6.86 mm. We attached an Apexel 10X Macro Lens (100 mm) to its rear camera to enhance the iPhone’s macro photography capabilities. Figures 5b, 5c, and 5ddisplay images of a *Hyalomma marginatum* sample captured using these cameras, in the order mentioned above. We set up our system (Fig. 5a) to capture high-resolution, close-up images of ticks with exceptional detail using different devices and optical configurations. This dataset includes multiple images per specimen, which may introduce sample dependence but improves exposure to intra-sample visual variability. This dataset is used to improve the reliability and applicability of our machine learning models.

#### Dataset from the Swedish Veterinary Agency (SVA)

The first confirmed instances of adult specimens of *Hyalomma marginatum* and *H. rufipes* in Sweden were reported between 2018 and 2019^44^. This significant discovery prompted the SVA to initiate an approach to monitor the potential spread of these exotic tick species. In 2019, the SVA launched a public awareness campaign encouraging individuals to submit images of exotic tick species via email. Togheter with the campaign a website at SVA:s homepage was set up with images and information on the tick species that was off high interest. In addition to image submissions, physical tick specimens of particular interest were sent to SVA, where experts conducted thorough morphological examinations, followed by pathogen analysis.

The dataset resulting from this initiative comprises 555 images, with some images representing the same tick sample from different angles or magnifications. The distribution of species within this dataset is depicted in table 1. This dataset has been partitioned into training, validation, and in-distribution test sets to support the development and evaluation of the machine learning model. The diverse nature of the images, including varying perspectives and quality, presents a realistic challenge for model generalization and robustness in identifying and classifying tick species.

#### Web application dataset

As detailed in the introduction, SVA launched a web application, ‘Report Tick,‘ on May 4, 2023, as part of a citizen science initiative to monitor tick findings across Sweden ^7^. This effort resulted in the collection of 15,885 tick reports, with over 99% of the identified ticks being classified as *Ixodes ricinus*. All labels were provided by experts at SVA. The distribution of the collected data is summarized in Table 1. Although *Ixodes persulcatus* was excluded from the classification task, some samples submitted from northern regions of Sweden may belong to this species due to its geographical overlap. These cases are likely misclassified as *I. ricinus* due to the high visual similarity between the two species.

**Table 1:** Distribution of tick species images across different data sources, including total counts per species and per source. Multiple images may correspond to the same individual tick.

Species	GBIF	SVA	TickExpand	Web application	Total
*Carios vespertilionis*	94	8	0	41	144
*Dermacentor reticulatus*	1177	0	451	9	1637
*Haemaphysalis punctata*	37	4	110	10	161
*Hyalomma marginatum*	133	3	441	9	586
*Ixodes hexagonus*	77	0	283	52	412
*Ixodes ricinus*	1003	540	0	15756	17299
*Rhipicephalus sanguineus*	432	0	927	8	1367
Dataset usage	Training/testing	Training/testing	Training	OOD testing	
Total	2954	555	2212	15,885	21,606

Among the datasets used, TickExpand is the only dataset containing preserved (frozen) specimens. Other datasets, including Web App and SVA submissions from the public, contain only fresh samples. While GBIF may include preserved specimens, specific preservation details were not systematically recorded.

**Table 2:** Distribution of attributes across tick species and datasets. The table reports counts of samples across three axes: Life Stage (Larva, Nymph, Adult, and Unknown), Sex (Female, Male, Multiple or Not applicable, and Unknown), and Feeding Status (Engorged, Not Engorged, and Unknown). Note that the same tick may appear in multiple images. “TickExpand” is a curated dataset; “Web App” contains public submissions; “SVA” is a research-grade dataset with limited annotation coverage.

		Life stage				Sex				Feeding		
Species	Dataset	L	N	A	Unk.	F	M	Mul	Unk.	Eng.	Non.	Unk.
*Carios vespertilionis*	TickExpand	N/A				N/A				N/A		
	Web App	0	0	18	7	2	15	1	7	1	17	7
	SVA	N/A				0	0	0	4	0	4	0
*Dermacentor reticulatus*	TickExpand	0	0	3	0	0	3	0	0	0	3	0
	Web App	0	0	6	1	6	0	0	1	0	6	1
	SVA	N/A				N/A				N/A		
*Haemaphysalis punctata*	TickExpand	0	0	1	0	1	0	0	0	0	1	0
	Web App	0	0	8	0	6	2	0	0	1	7	0
	SVA	N/A				1	0	0	0	0	1	0
*Hyalomma marginatum*	TickExpand	0	0	3	0	0	3	0	0	0	3	0
	Web App	0	0	5	0	3	2	0	0	2	3	0
	SVA	N/A				1	1	0	0	0	2	0
*Ixodes hexagonus*	TickExpand	1	3	0	0	2	2	0	0	1	2	0
	Web App	1	1	36	0	38	0	0	0	36	1	1
	SVA	N/A				N/A				N/A		
*Ixodes ricinus*	TickExpand	N/A				N/A				N/A		
	Web App	13	788	11668	101	10316	1557	562	135	7166	5270	134
	SVA	N/A				266	29	10	10	145	165	7
*Rhipicephalus sanguineus*	TickExpand	1	6	0	0	3	4	0	0	0	7	0
	Web App	0	1	3	0	3	0	0	1	2	2	0
	SVA	N/A				N/A				N/A		

Table 2 presents the distribution of life stage, sex, and feeding status across species and datasets, including data from the Web App, TickExpand, and SVA datasets. Since this information was unavailable for the GBIF dataset, it was excluded from the table. Note that the counts reflect individual tick samples, not images—multiple images may exist for a single sample, resulting in lower numbers than the total number of images.

### Preprocessing of the data

To utilize pre-trained deep learning models, we standardized all images to a resolution of 512x512 pixels. Before resizing, we employed an object detection model based on the InceptionResNetV2 architecture, which was trained on ImageNet and fine-tuned with a Faster R-CNN head on the OpenImages V4 dataset, which contains 600 classes. This model, available through the TensorFlow Object Detection API ^45^, was applied to each image in the dataset.

**Fig. 6:**
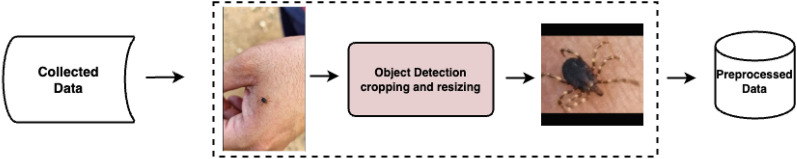
Data preprocessing example.

For each image, if the object detection model identified any of the following labels: Tick, Insect, Bug, Beetle, Invertebrate, Isopod, Centipede, Ladybug, Ant, Spider, or Snail, and the detected object occupied less than 10% of the total image area, we cropped the image to the bounding box of the detected object (Fig. 6). We tested this approach by conducting a CNN classification with varying thresholds for the object occupation percentage and simply no cropping. The results indicated that using a 10% threshold yielded the most significant improvement in classification accuracy, validating the effectiveness of this preprocessing step.

To prevent data leakage across training, validation, and in-distribution test sets, we ensured that images from the same sample were grouped together and not split across different sets. This was particularly important given the presence of multiple images per sample in our datasets. For the limited video data we obtained from the SVA and web application datasets, which typically featured short clips with consistent lighting and positioning, we randomly selected a single frame from each video to include in the dataset.

After completing these preprocessing steps, we allocated 70% of the GBIF and SVA datasets for training, 10% for validation, and 20% for in-distribution testing. Due to the limited number of samples in the TickExpand dataset, all of its data was used only for training. As mentioned previously, the web application dataset was exclusively reserved for out-of-distribution testing.

### Model architectures

Transfer learning is a machine learning technique that involves repurposing a pre-trained model, initially developed for one task, as the starting point for a model on a different but related task. This methodology leverages the knowledge embedded in the pre-trained model, typically derived from a large-scale dataset, and applies it to another dataset that is often smaller or domain-specific. The effectiveness of transfer learning has been well-documented in the literature, particularly in the image classification domain, where fine-tuning pre-trained models on new datasets has been shown to significantly enhance model accuracy and efficiency ^46–48^. In this study, we experimented with three state-of-the-art deep learning architectures: ResNet152V2 ^49^, InceptionResNetV2 ^50^, and EfficientNetV2M ^51^. The choice of these architectures was motivated by their proven performance in various computer vision tasks and their ability to leverage transfer learning for improved generalization on new datasets effectively.

Out of the three architectures that were tested, EfficientNetV2M showed improvements in classification accuracy and generalization, surpassing the other models. This indicates that the architecture’s advanced design, scalability, and fine-tuning process make it especially suitable for the task at hand.

### Training procedure

Data augmentation ^52^ is an important technique used in training machine learning models, especially for tasks like image classification. This technique involves applying random transformations to the input data, effectively increasing the diversity of the training set without requiring additional storage space. Here, we used a variety of augmentation strategies, such as rotation, shifting, flipping, zooming, and adjusting brightness. These transformations were applied dynamically in real-time during the training process, ensuring that each batch presented a unique variation of the data to the model and engaging the model in a dynamic learning process.

This method helps the model to generalize to unseen data by preventing it from becoming overly focused on the specific characteristics of the training set. Continuous exposure to varied versions of the data encourages the model to learn more robust and generalized features, ultimately leading to improved performance across diverse test scenarios.

The training process for our model was conducted in two distinct stages, each designed to optimize different aspects of the learning process. In the initial stage, we employed pre-trained models in a “frozen” state, where the parameters of the pre-trained layers were kept fixed and untrainable. This stage focused on training a custom classification head, which included a GlobalAveragePooling layer, a Dropout layer, and a fully connected output layer with seven nodes corresponding to the target classes. The AdamW optimizer ^53^ was utilized for optimization, with an initial learning rate set to 0.001. To address the class imbalance inherent in the dataset, we incorporated the Categorical Focal Cross entropy loss function ^54^, supplemented with label smoothing at 10% and an alpha parameter to reflect class weights.

We have used early stopping and a learning rate reduction strategy to improve training stability and prevent overfitting. These techniques are essential for preventing the model from being stuck in local minima and ensuring it can perform well beyond the training data. The batch size was fine-tuned to 32, balancing computational efficiency and model performance.

After completing the first stage of training, we proceeded to the fine-tuning phase. In this second stage, we unfroze the last 40 layers of the pre-trained model, allowing these layers to be trained and fine-tuned to the specific features of our target dataset. The fine-tuning stage used the same hyperparameters as the initial stage, except for a reduced learning rate set to 0.0001. This lower learning rate was chosen to adjust the pre-trained weights without significantly changing the model’s learned representations, thereby enhancing its adaptation to the specific characteristics of the new data.

Using data augmentation and a two-stage training process, we created a robust, well-generalized model that accurately classifies target classes despite significant data variations. The models and image generation pipelines were developed using TensorFlow 2.16.1, an open-source Python package. Each model was retrained three times with different initializations, and results are reported as mean ± standard deviation across runs using the same test set.

### Model evaluation

In the context of public and animal health, failing to identify a tick species (false negative) poses a greater risk than a false positive classification. High recall is, therefore, especially critical, as it ensures that rare or exotic species are detected—even at the cost of occasional misclassification. This supports the early-warning function of our surveillance system and motivates the metrics we prioritize in our evaluation.

To evaluate the performance of our classification models, we used metrics suited to multiclass classification tasks with highly imbalanced datasets. Given the extreme class imbalance in our data, relying solely on standard accuracy or weighted averages can obscure poor performance in underrepresented classes. Therefore, we adopted a multi-perspective evaluation strategy that includes both class-specific and aggregate metrics.

We computed precision, recall, and F1-score for each class to capture detailed, class-level performance. Precision quantifies the correctness of positive predictions for a given class, while recall (sensitivity) reflects the ability to detect all true instances of that class. The F1-score, being the harmonic mean of precision and recall, balances the two — and is particularly valuable in highlighting the trade-offs between false positives and false negatives.

To summarize class-level metrics, we computed both macro and weighted averages. Macro-averaged metrics treat each class equally and are critical for evaluating fairness and robustness across species. Weighted averages, on the other hand, reflect class frequencies and are dominated by the performance of majority classes such as *Ixodes ricinus*, which can be misleading under imbalance.

Given this, we intentionally prioritize macro-averaged recall (equivalent to balanced accuracy), macro F1-score, and the Matthews Correlation Coefficient (MCC) for our primary evaluations. We deliberately chose not to emphasize macro-averaged precision, as it can be heavily skewed by small sample sizes in rare classes, leading to unstable or overly optimistic values that do not reflect consistent model behavior. In highly imbalanced settings, macro precision may fluctuate significantly due to a few false positives or negatives in underrepresented classes and thus offers limited reliability compared to recall-based measures. By focusing on macro recall, macro F1, and MCC, we aim to capture a more balanced and class-independent view of performance — particularly for rare but ecologically significant species. These metrics provide more reliable indicators of true classification ability across all classes. The MCC is well-suited for imbalanced classification tasks, as it integrates information from all four confusion matrix components—true and false positives and negatives—into a single performance metric ranging from -1 to +1. ^55^.

Although we report Area Under the ROC Curve (AUC-ROC) for completeness, we recognize that it has limitations in multiclass and imbalanced contexts. AUC is based on ranking probabilities, not on hard predictions, and may appear high even if a model systematically misclassifies minority classes. Therefore, while we include macro AUC, it is not emphasized in our core evaluation.

To provide a clear view of class-specific behavior, we present both the raw (unnormalized) and row-normalized confusion matrices. The unnormalized version shows absolute classification counts, while the normalized version makes it easier to interpret per-class recall, as each row is scaled to sum to one. This dual presentation helps interpret whether performance issues arise from false positives, false negatives, or both — particularly for rare classes where raw counts may be misleading.

To robustly assess the statistical reliability of these metrics, particularly given the small sample sizes in some classes, we calculated 95% confidence intervals using non-parametric bootstrapping. Specifically, we resampled test data with replacement for 1000 iterations, generating distributions for precision, recall, F1-score, and MCC. These distributions enabled us to estimate the variability and uncertainty in performance metrics. Classes with fewer than 10 test samples are flagged explicitly, as their wide confidence intervals indicate high uncertainty, cautioning against strong interpretations of performance in these cases.

In summary, our evaluation framework is designed to avoid being biased by class frequencies and instead highlight the true strengths and weaknesses of the model across all tick species.

## Results

### In-distribution test results

Here, we present the performance of these models on the in-distribution test data, as outlined in the Subsection Data Sources. The results for three selected models—ResNet152V2, InceptionResNetV2, and EfficientNetV2M—are shown in Table 3, which summarizes their macro recall, F1-score, AUC and MCC metrics.

**Table 3:** In-distribution test results for three models.

Model	Macro recall	Macro F1-score	Macro AUC	MCC
ResNet152V2	0.68 ± 0.2	0.66 ± 0.1	0.82 ± 0.1	0.64 ± 0.1
InceptionResNetV2	0.67 ± 0.3	**0.68** ± **0.3**	0.84± 0.2	0.67 ± 0.2
EfficientNetV2M	**0.72 ± 0.3**	0.67 ± 0.1	**0.85** ± **0.1**	**0.71** ± **0.1**

Significant values are in bold.

From Table 3, we observe that the three models exhibit very close performance, with EfficientNetV2M slightly outperforming the others, particularly regarding recall. This is an important metric for our study, where correctly identifying true positives is critical. The higher AUC score of EfficientNetV2M further highlights its ability to distinguish between classes effectively. To ensure the robustness of our results, we retrain each model three times using the same dataset but with different random initializations. The reported values in the results tables represent the mean performance across these runs, while the ± values indicate the standard deviation.

**Fig. 7:**
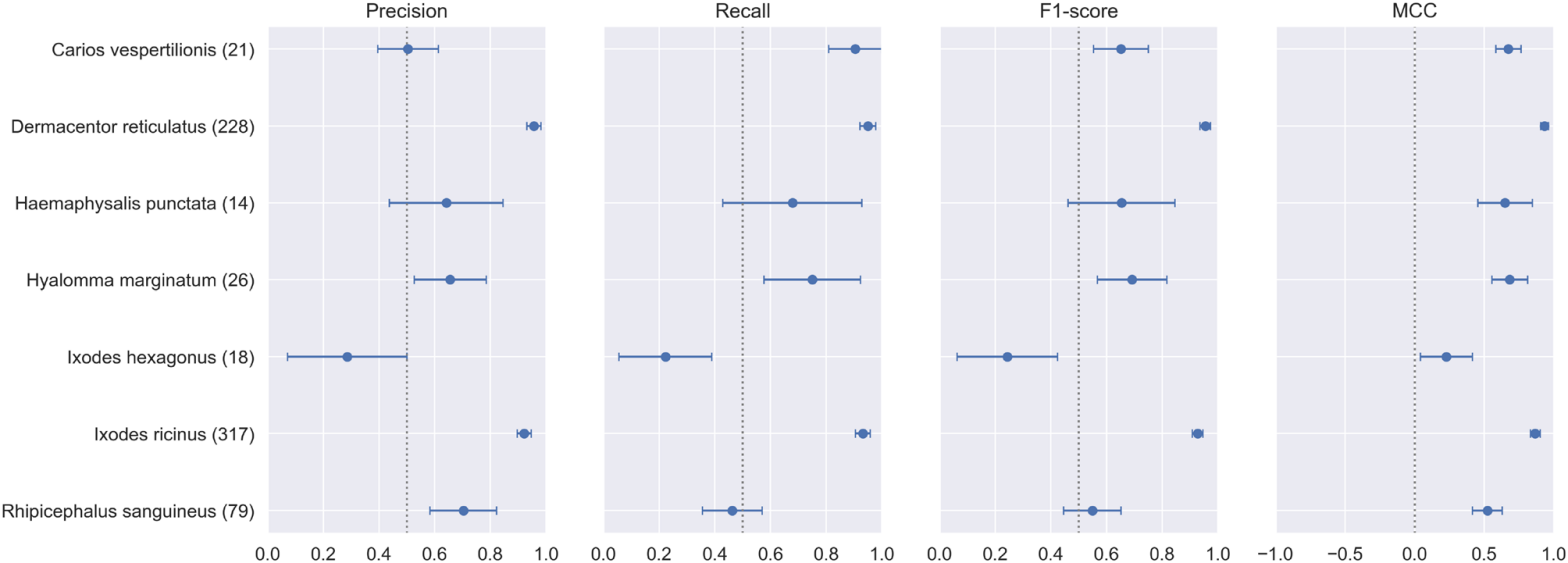
Per-class precision, recall, F1-score, and MCC on the in-distribution test set. Points represent means, horizontal bars denote 95% bootstrap confidence intervals (1000 resamples), and parentheses indicate the number of test samples per class.

**Fig. 8:**
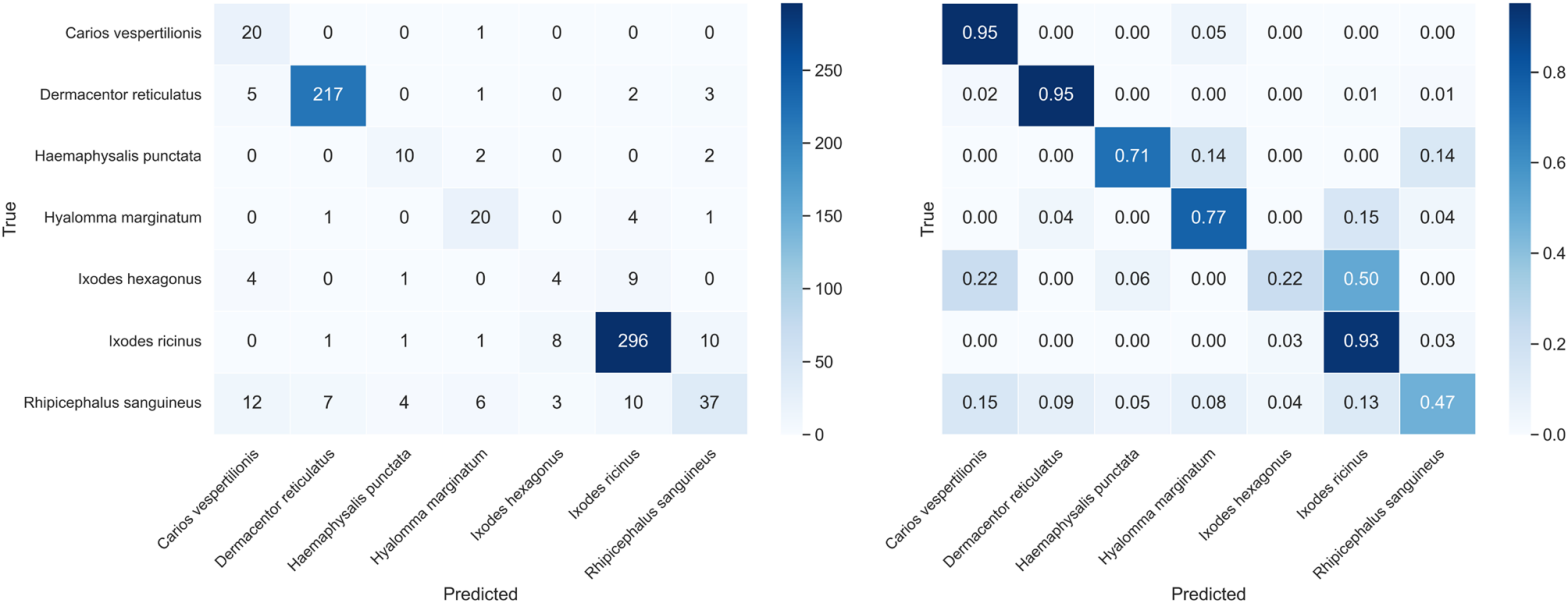
In distribution test confusion matrix from EfficientNetV2M.

To gain a more detailed understanding of the EfficientNetV2M model’s performance, we have included the confusion matrix in Fig. 8 and per-class precision, recall, F1-score, and MCC along with their confidence intervals in Fig. 7. Two species, *Ixodes hexagonus* and *Rhipicephalus sanguineus*, show notably poor performance with wide confidence intervals, indicating high uncertainty and frequent misclassification. In the case of *Ixodes hexagonus*, many samples are misclassified as *Ixodes ricinus* (50%), suggesting that the model struggles to distinguish between these species, likely due to imbalance of the training datasets. Similarly, *Rhipicephalus sanguineus* exhibits lower recall (47%) with many misclassifications across various species. These factors, along with the impact of this species’ appearance on the prediction outcomes, will be further explored in the discussion section.

Overall, EfficientNetV2M demonstrates better results across most species, with higher overall MCC and AUC, making it a promising approach for large-scale tick classification.

### Out-of-distribution test results

The web application dataset, which consists of purely out-of-distribution (OOD) data, was used for the final testing to evaluate the model’s generalizability. The results for each of the models are shown in Table 4, which presents macro recall, F1-score and AUC, and MCC for each model.

**Table 4:** OOD test results for three models. The results are reported as the mean and standard deviation over three runs.

Model	Macro recall	Macro F1-score	Macro AUC	MCC
ResNet152V2	0.42 ± 0.2	0.20 ± 0.1	0.71 ± 0.2	0.33 ± 0.3
InceptionResNetV2	0.49 ± 0.3	0.25 ± 0.2	0.72 ± 0.2	0.425 ± 0.3
EfficientNetV2M	**0.55** ± **0.3**	**0.27** ± **0.2**	**0.75** ± **0.2**	**0.49** ± **0.2**

Significant values are in bold.

EfficientNetV2M showed relatively strong performance in terms of recall and F1-score on the OOD data, indicating a promising ability to generalize to novel distributions. Additionally, EfficientNetV2M obtained the highest MCC, suggesting a better overall agreement between predicted and true labels compared to the other models, even in out-of-distribution scenarios.

**Fig. 9:**
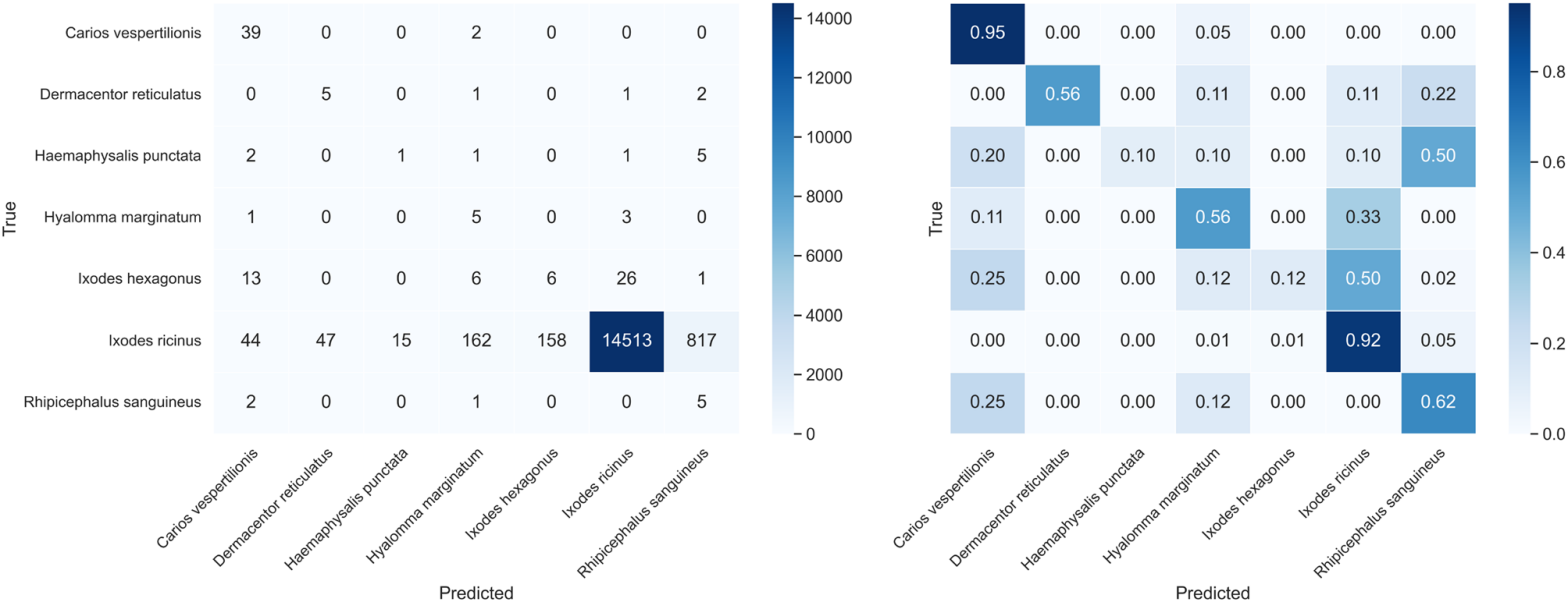
Confusion matrix for EfficientNetV2M on out-of-distribution test data.

The confusion matrix for EfficientNetV2M on OOD data is displayed in Fig. 9. The model shows variability in its predictions, with some species being more difficult to classify.

Table 5 reports the class-wise performance of the EfficientNetV2M model on the out-of-distribution (OOD) test set. The table includes precision, recall, F1-score, and support for each class, as well as overall accuracy, macro-averaged, and weighted-averaged metrics. Performance varies across classes, reflecting the distribution and characteristics of the test data.

**Table 5:** Detailed classification metrics per class on the real-world, out-of-distribution test set evaluated with the EfficientNetV2M model.

Class	Precision	Recall	F1-score	Support
*Carios vespertilionis*	0.39	0.95	0.55	41
*Dermacentor reticulatus*	0.10	0.56	0.16	9
*Haemaphysalis punctata*	0.06	0.10	0.08	10
*Hyalomma marginatum*	0.03	0.56	0.05	9
*Ixodes hexagonus*	0.04	0.12	0.06	52
*Ixodes ricinus*	1.00	0.92	0.96	15756
*Rhipicephalus sanguineus*	0.01	0.62	0.01	8
Accuracy			0.92	15885
Macro average	0.23	0.55	0.27	15885
Weighted average	0.99	0.92	0.95	15885

#### Evaluation with average probabilities for each sample

In the web application dataset, some samples consist of multiple images. To make use of all available information, the model predictions were averaged across all images belonging to the same sample. This technique was applied in order to improve the prediction consistency across multiple images of the same tick sample. The sample distribution before and after filtering for unique samples is shown in Table 6.

**Table 6:** Sample distribution before and after filtering unique samples.

Species	Original Distribution	Unique samples
*Carios vespertilionis*	41	25
*Dermacentor reticulatus*	9	7
*Haemaphysalis punctata*	10	8
*Hyalomma marginatum*	9	5
*Ixodes hexagonus*	52	38
*Ixodes ricinus*	15756	12570
*Rhipicephalus sanguineus*	8	4

Applying average probabilities across multiple images per sample improved recall and MCC, suggesting enhanced consistency in predictions when more contextual views are available. The EfficientNetV2M model, in particular, achieved a recall of 0.60 and an MCC of 0.55, both of which represent improvements compared to the previous results (Table 7).

**Table 7:** OOD test results with average probability for each sample.

Model	Macro Recall	Macro F1-score	Macro AUC	MCC
ResNet152V2	0.43 ± 0.3	0.21 ± 0.1	0.73 ± 0.2	0.34 ± 0.2
InceptionResNetV2	0.48 ± 0.3	0.23 ± 0.2	0.76 ± 0.2	0.43 ± 0.1
EfficientNetV2M	**0.60** ± **0.3**	**0.26** ± **0.2**	**0.78** ± **0.2**	**0.55** ± **0.1**

**Fig. 10:**
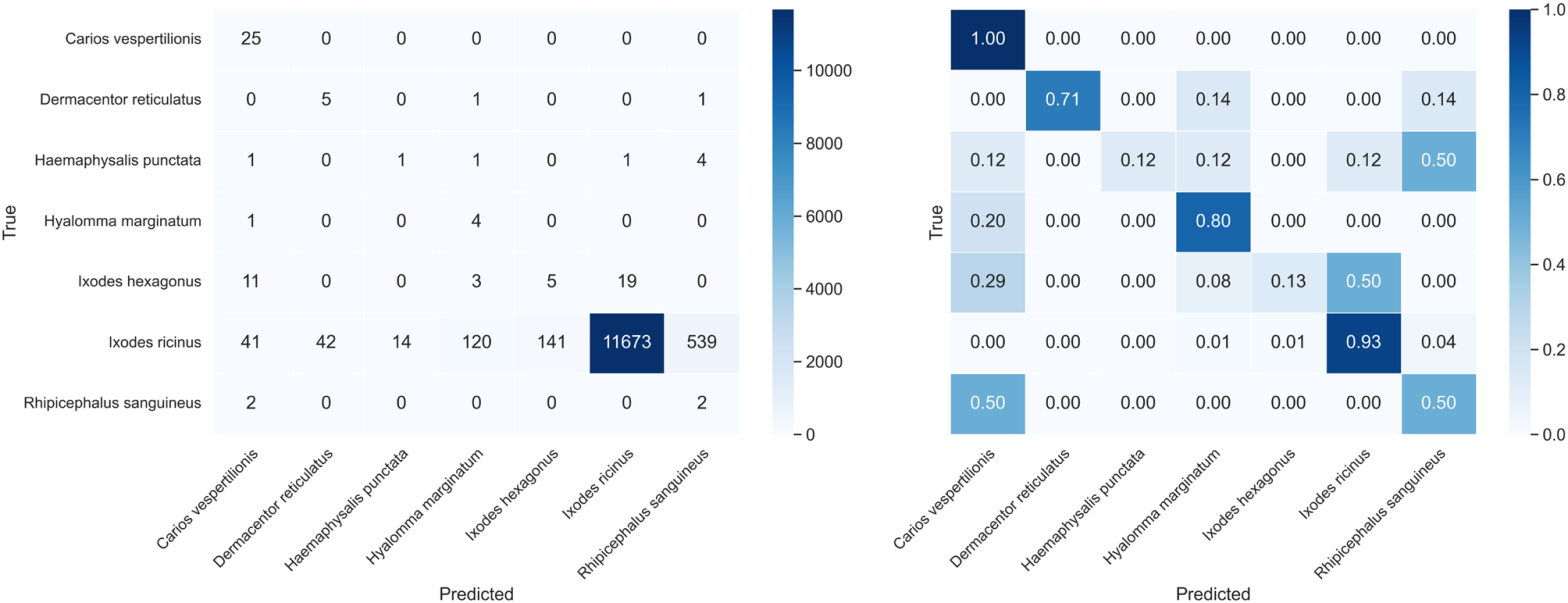
Confusion matrix for OOD test data with average probability for each sample on the trained EfficientNetV2M.

The confusion matrix after averaging probabilities for each sample is shown in Figure 10. The averaging approach reduces misclassification by improving the model’s ability to make more accurate predictions when multiple images of the same sample are provided.

**Fig. 11:**
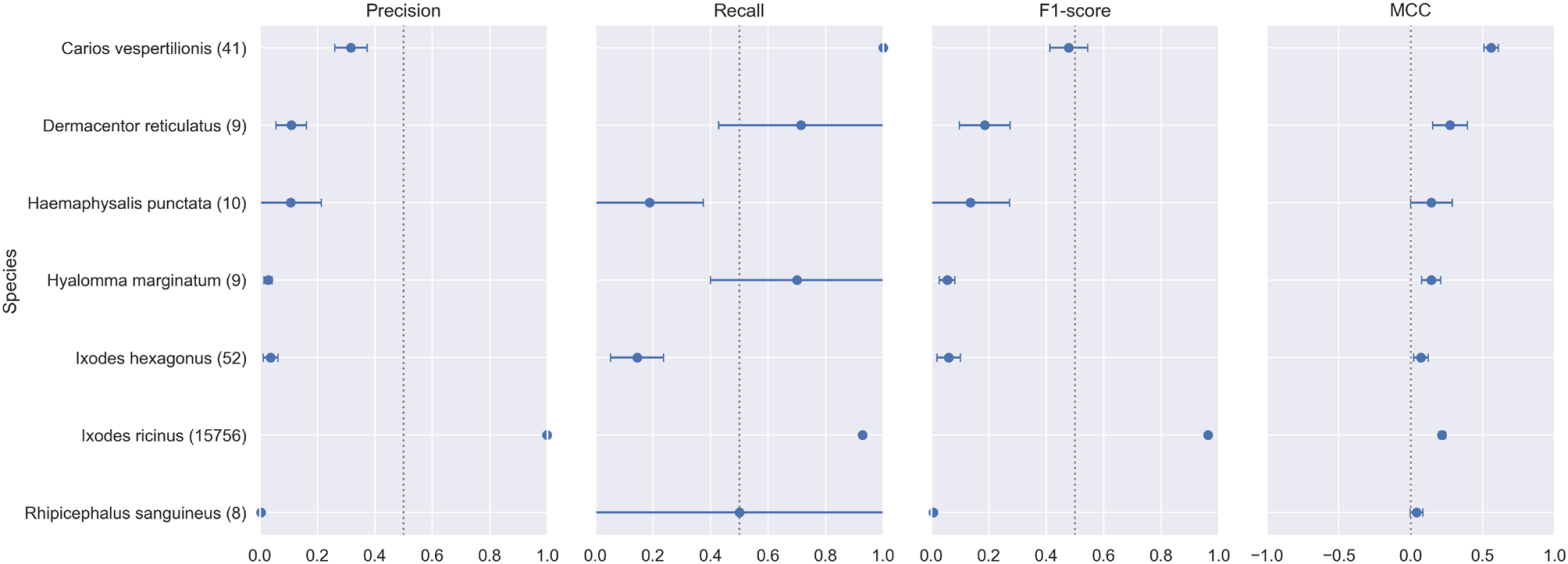
Per-class precision, recall, F1-score, and MCC on the out-of-distribution test set using averaged probabilities per sample. Points represent means, bars denote 95% bootstrap confidence intervals (1000 resamples), and parentheses indicate the number of unique test samples per class.

The confidence intervals (CIs) shown in Figure 11 further illustrate the model’s uncertainty in predicting rare species in the out-of-distribution (OOD) dataset. While *Ixodes ricinus* maintains stable and reliable performance (narrow CIs), classes like *Ixodes hexagonus*, *Rhipicephalus sanguineus*, and *Hyalomma marginatum* show notably wide intervals, particularly for recall and MCC. This wide uncertainty highlights significant challenges in reliably classifying underrepresented species and underscores the importance of interpreting these results cautiously.

We present sample-level predictions in the context of both correctly classified and misclassified examples. For each sample, the top three predicted probabilities were used to provide insights into the model’s decision-making process.

**Fig. 12:**
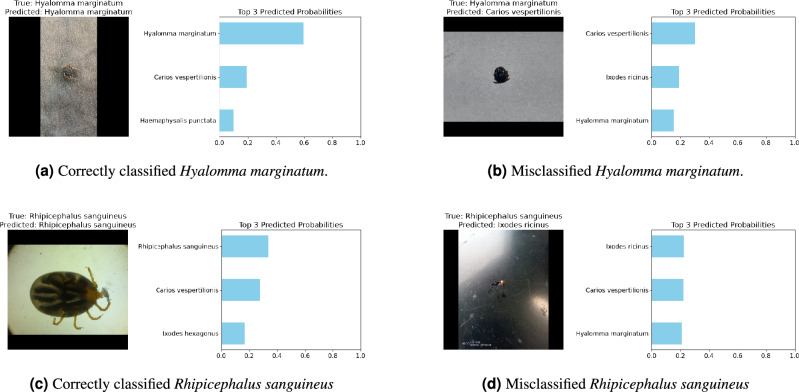
Examples of correctly classified and misclassified samples, showing the top three predicted probabilities.

Figure 12 highlights some instances of correctly classified and misclassified samples. Each subfigure shows a correctly or misclassified tick image along with its top three predicted probabilities. For example, Fig. 12adisplays a correctly classified *Hyalomma marginatum* sample, while Fig. 12bshows a misclassified example of the same species.

These visualizations provide insight into the model’s classification process and can guide future improvements.

### Evaluating model interpretability through explainable AI

We integrated Explainable AI (XAI) techniques into our evaluation framework. Specifically, we employed the Randomized Input Sampling for Explanation (RISE) method ^18^, which is designed to provide a visual and intuitive understanding of the model’s predictions. RISE generates saliency maps highlighting the most critical regions of the input image that influenced the model’s final classification. These maps provide insights into which parts of the image the model “looked at” when making its decision, thereby making the deep learning model more interpretable.

RISE operates by applying random masks to the input image and observing the changes in the model’s prediction. By aggregating these results over many trials, RISE constructs a saliency map that assigns higher importance to regions that consistently contribute to the model’s prediction. This process allows us to visualize the decision-making patterns of the EfficientNetV2M model, revealing whether the model relies on meaningful features such as specific morphological traits of the ticks or irrelevant background information.

The RISE Insertion Score reflects the model’s reliance on specific image regions by gradually inserting the most important pixels back into the image and measuring the corresponding increase in the model’s confidence. Higher insertion scores indicate that the model is correctly focusing on the relevant features when making predictions.

**Fig. 13:**
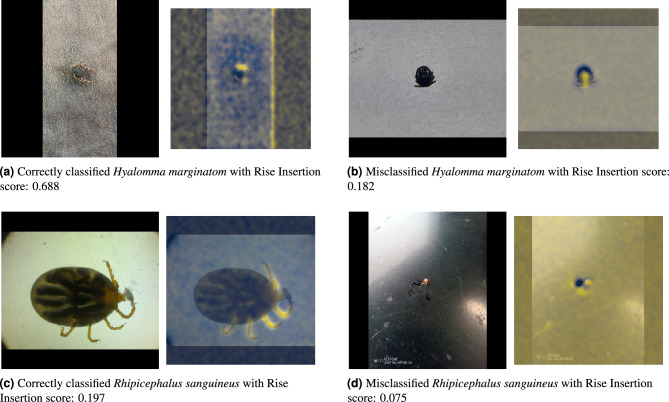
Examples of saliency maps generated by the RISE method for tick species classification using the EfficientNetV2M model.

In Fig. 13, we provide examples of saliency maps generated by the RISE method for various classifications by the EfficientNetV2M model. The figure includes instances of both correctly and incorrectly classified species, highlighting the difference in model focus between successful and failed predictions.

In the analysis of the saliency maps generated by the RISE method, the results demonstrate varied model performance across different classifications. For instance, in the case of *Hyalomma marginatum*, the model correctly classified the species in one instance with a high RISE Insertion score of 0.688. The corresponding saliency map highlighted regions containing critical morphological features of the species, indicating that biologically relevant traits influenced the model’s decision. On the other hand, in a different case, the model incorrectly identified *Hyalomma marginatum* and received a lower RISE Insertion score of 0.182. The saliency map showed that the model’s focus was spread across irrelevant areas of the image, which probably led to the wrong classification.

## Discussion

In machine learning, particularly in high-stakes applications such as medical diagnostics, environmental monitoring, and wildlife classification, the transparency and interpretability of model predictions are paramount. While state-of-the-art models, such as deep neural networks, achieve high accuracy, they often operate as black boxes, making it difficult to understand how predictions are made. This lack of interpretability can be problematic, especially in scenarios where trust and validation of the model’s decision-making process are crucial. Our findings suggest that model attention plays an important role in accurate classification, especially when dealing with species that show small morphological differences. All presented findings and challenges highly contribute to the development of automated tools that can assist researchers and the animal and public health sector in monitoring and responding to the evolving threat of tick-borne diseases.

The performance of the trained models in both in-distribution and OOD scenarios reveals several key challenges in deploying AI for tick classification. While EfficientNetV2M achieved higher overall performance primarily due to *Ixodes ricinus*, it showed limited performance across most other species in the OOD set, highlighting the challenges of generalization under extreme class imbalance. This is likely due to the species similarity challenge, where small morphological differences between species make accurate classification difficult, even for models trained on large datasets.

One of the major challenges we faced was the lack of data, especially for species such as *Haemaphysalis punctata* and *Carios vespertilionis*, for which fewer samples were available for training. This unequal data distribution resulted in models biased towards the majority species, *Ixodes ricinus*. In order to counter this, we used the data augmentation technique and categorical focal loss with class weights for the model training process. However, the limited number of samples for rare species still prevents the model from generalize well. We also experimented with several versions of Data-efficient Image Transformers ^56^ and ConvNeXT ^57^, which leverage transformer-based architectures but likely due to the extremely small sample size of certain species; these models did not yield satisfactory results. To address this issue, additional data collection is necessary, particularly for underrepresented species and for engorged ticks, which were not well-represented in the dataset.

Beyond data scarcity, we observed specific overfitting to *Carios vespertilionis*, likely driven by limited variability in training samples. This overfitting was reflected in the confusion matrix, where the model showed unusually high recall in classifying *Carios vespertilionis*. This likely resulted from the species’ distinct morphological features and the fact that all the images in the dataset represented adult stages, leading to a lack of variability. The uniformity in the images, combined with its unique appearance, may have made it easier for the model to recognize, resulting in almost perfect recall. However, this suggests that the model might be relying too heavily on the specific visual traits of adult ticks and may struggle with generalizing to other life stages or similar-looking species, indicating the need for a more diverse dataset to improve robustness. The supplementary information includes a saliency map example for *Carios vespertilionis*.

Exact species identification is not a realistic goal at this point, especially in cases where two tick species from the same genus are extremely similar (e.g., *I. ricinus* and *I. persulcatus*; *H. marginatum* and *H. rufipes*; *D. reticulatus* and *D. marginatus*). For these species, we can often only identify up to the genus level. However, this level of identification may be sufficient to support monitoring of endemic tick spread and the detection of exotic species in Sweden, although further validation is needed to confirm its practical utility in real-world surveillance. Particularly for exotic ticks, reporters have the opportunity to send specimens to SVA for further morphological identification and pathogen analysis. On the other hand, species with more similar morphological traits, such as *Ixodes hexagonus* and *Rhipicephalus sanguineus*, exhibited higher misclassification rates, highlighting the challenge of distinguishing visually similar species. This misclassification is particularly evident in the case of *Ixodes hexagonus*, where nearly 50% of samples are predicted as *I. ricinus* in the OOD test set (Figure 10). Since 36 out of 38 *I. hexagonus* samples are labeled as engorged adults (Table 2), and the stratified results (Supplementary Table S2 and S3) show very low recall for this class under the engorged condition, it is likely that feeding status contributes substantially to this confusion. Morphological distortion caused by engorgement may obscure key species-specific traits, especially between closely related taxa like *I. ricinus* and I. *hexagonus*. This reinforces the need for a hierarchical classification approach, where engorgement status is first predicted as a separate step, followed by species-level identification within each morphological state, as proposed in our future work, which is further detailed below. This suggests that when visual differences between species are sufficiently distinct and preserved across conditions, the model can generalize well even with limited training data. However, for visually similar species, particularly when morphological features are obscured by factors like engorgement, the model may require more diverse and abundant examples to achieve reliable discrimination.

Intra-species variability across life stages further complicated classification. As seen in Figure 4, the morphological characteristics of ticks vary significantly between their larval, nymphal, and adult stages, as well as between engorged and non-engorged individuals. This morphological diversity often caused the models to confuse ticks in one stage with those in another, particularly when the model had limited exposure to examples from all life stages during training. Specifically, the dataset for *Haemaphysalis punctata* is largely dominated by images derived from a single individual (110 of 161 images), collected through the TickExpand dataset. This lack of variability clearly contributed to the poor recall (10%) observed for this species in the out-of-distribution evaluation. While this overrepresentation limits morphological diversity and generalization, training on duplicated individuals also has advantages. The TickExpand dataset provides high-quality, standardized images across varying angles and lighting, simulating the heterogeneity seen in citizen submissions. This may help stabilize feature extraction in early training and compensate for class scarcity where field data are unavailable. Neverdeless, future efforts should focus on expanding the dataset to include more diverse representations of each species across their life stages.

Another critical challenge we identified was the impact of tick engorgement on classification accuracy. Engorged ticks exhibit dramatically altered morphological characteristics compared to non-engorged ticks, often obscuring key species-specific features and consequently leading to misclassifications. An optimal solution could involve adopting an eight-class classification approach, comprising the seven species plus an additional separate class specifically for engorged ticks, or even employing a hierarchical classification model. Such a hierarchical model would first classify ticks based on their engorgement status, followed by a species-level classification within each category. However, due to the lack of labeled engorgement status in a significant portion of our training dataset, implementing this method has not been feasible in the current study. While Table 2 summarizes feeding status annotations from curated datasets, it does not include the GBIF dataset, where feeding status was not labeled by experts. Nevertheless, visual inspection indicates that engorged individuals are likely present across most species in GBIF. These images were retained in the training process to preserve class diversity, especially for underrepresented species, though the lack of explicit annotations limited our ability to stratify or control for engorgement effects during model development. Nonetheless, as the citizen science initiative is ongoing, we have received additional datasets from subsequent data collection phases through the web-based platform, where the engorgement status has been systematically labeled by experts. Our future plan involves leveraging this labeled dataset from the first and second years of citizen science participation to train a dedicated engorgement detection model. We anticipate that this separation and initial identification of engorgement status will significantly enhance the overall accuracy and reliability of species classification, dramatically improving model predictions in real-world scenarios.

Another challenge is posed by the fact that juvenile stages lack some species-specific morphological characters/features, complicating identification at the genus or species level. Direct examination under a stereomicroscope and/or high-quality images (which average citizens cannot typically produce in field conditions) is currently required for juvenile identification. Moreover, molecular identification is sometimes necessary. Since most citizen science data consist of adult and engorged stages, with very few nymphs and almost no larvae, it is currently more practical to focus on identifying adult stages at the genus or species level. For this reason, the goal for AI-based identification of juveniles should be set at the developmental stage level (i.e., larva and nymph). A similar challenge arises with the engorgement of different developmental stages, which can obscure key features necessary for species identification. In this case, a realistic goal could be to classify such specimens simply as “engorged ticks.”

Since the size of the specimen is also a critical feature that enables a correct identification, in the future development of the proposed technique, we have to face this challenge. For example, AI-based techniques that already became commercially available for the identification of helminth eggs (e.g., Ovacyte - Telenostic, Imagyst-Zoetis, Micron kit from Micron Agritech) did not have to face this problem. In these systems, the camera capturing the microscopic images is also able to measure the object size.

The OOD test results provide valuable insights into the model’s ability to generalize to unseen data, which is critical for real-world applications where citizen-submitted data varies significantly in quality and conditions. The model’s performance on the OOD data notably decreased compared to the in-distribution results, particularly affecting macro F1-score and precision. Averaging predictions over multiple images per sample did help mitigate some misclassification, improving the MCC and macro recall. Nevertheless, the model’s robustness was tested by the wide range of images submitted by citizens, with quality from high-resolution, well-lit professional images to low-resolution and inconsistent submitted photos, which emphasized the need for further fine-tuning based on specific data qualities to handle diverse data sources effectively. The statistical reliability of model performance for certain species, notably *Dermacentor reticulatus*, *Hyalomma marginatum*, and *Rhipicephalus sanguineus*, is limited due to small sample sizes in the test dataset. For these species, the limited sample sizes (e.g., nine for *Dermacentor reticulatus*) produce wide confidence intervals, resulting in substantial uncertainty regarding performance metrics. To transparently illustrate this uncertainty, we computed 95% confidence intervals using non-parametric bootstrapping (see Figure 11), clearly highlighting the limited interpretability of these results. Additionally, the underlying dataset constraints, including severe class imbalance, overrepresentation of individual ticks (as seen in *Haemaphysalis punctata*), and variability due to feeding status, significantly undermine the model’s generalization capability. Addressing these dataset limitations through increased sample diversity and stratified evaluations will be critical for improving future model performance and reliability.

The quality of tick samples can cause complications for classification. Samples that have been frozen, preserved in alcohol, or physically manipulated during removal from their hosts can be damaged or deformed. This makes it difficult for AI to correctly classify species because images taken from these compromised samples often lose key distinguishing features. Therefore, integrating these images into the classification process requires careful consideration and may require specific preprocessing techniques to minimize the impact of these distortions on model performance.

Data leakage, a common issue in machine learning, occurs when information from the test set inadvertently influences the training process, leading to artificially inflated performance metrics. This can significantly compromise the generalization ability of the model in real-world situations. To mitigate this, we ensured that the TickExpand dataset was only used for training, with no overlap in the test sets. Additionally, we meticulously tracked the samples from the GBIF and SVA datasets, ensuring that repeated occurrences of the same samples were either entirely within the training or testing sets but never across both. This careful management of data sources helped prevent leakage and ensured the integrity of our evaluation process.

We have built on and extended previous work by implementing advanced image classification techniques to automate the detection and classification of tick species from citizen-submitted images. Despite the promising results, it is important to note that expert verification remains a critical step in the tick classification process. Given the variability in data quality and the potential for misclassification, the model’s predictions should be viewed as supportive tools rather than definitive results. Consistent communication with domain experts during the data collection process is also essential. For instance, the data collection efforts from GBIF were delayed due to the outdated taxonomy of *Carios vespertilionis*, previously known as *Argas vespertilionis*. This issue was further complicated by specific sources, such as iNaturalist, which still used the outdated name, leading to data retrieval and classification discrepancies. However, new findings from the present study will significantly contribute when working with large data sets from the ongoing citizen science project, both by saving time for the experts labeling and controlling the incoming images and by giving the reporter quick feedback. The latter will, in turn, hopefully increase the will to report a new finding. In this way, citizen science can generate detailed real-time data on species introduction and spread. Larger datasets enhance AI algorithms, leading to more effective strategies ranging from sampling techniques to mitigation efforts. More importantly, this process engages and educates the general public, fostering meaningful relationships between citizens and the scientific community.

The use of explainable AI (XAI) techniques, like the RISE method, has been proven practical in enhancing the transparency of the model’s decision-making process. By creating saliency maps, we can visualize the areas of the input images that had the most impact on the model’s predictions, thus increasing confidence in the system’s results. However, there are still challenges in ensuring that these saliency maps focus on biologically relevant features rather than background noise. The insertion metric applied in this study quantified the fidelity of the explanations, revealing that correctly classified instances showed a significant increase in prediction confidence when the most important pixels were added back into the image. There is an urgent need for further refinements to the XAI techniques to help increase trust and reliability in the model’s predictions, especially when used by non-expert end-users in citizen science initiatives.

Technological limitations related to the hardware and software used for capturing tick images also posed challenges. Ensuring that models are trained and validated on data representing the full diversity of tick images captured under various conditions is crucial for generalization. Moreover, ensuring the privacy and ethical considerations of citizen-submitted data is critical, especially as tick images may include sensitive metadata or identifiable features.

Additionally, the computational demands for optimizing these models, especially during extensive hyperparameter tuning, were considerable. Each combination of model structure and hyperparameters required high-performance computing resources, with extensive GPUs or TPUs access to efficiently process the large datasets and conduct multiple training runs.

In conclusion, the results demonstrate the potential of AI for tick classification. However, challenges such as data scarcity, species similarity, and image quality variability remain. Future work should prioritize expanding the dataset, particularly for rare species and life stages, improving model interpretability through XAI, and addressing the practical concerns of deploying AI in real-world situations. Rather than presenting this work as achieving robust classification across all species, we frame it as a proof-of-concept infrastructure for integrating AI with citizen science to support scalable, real-world tick surveillance. Continued integration with expert verification and feedback from citizen science initiatives will be crucial for ensuring the long-term success and reliability of these AI systems.

## Supplementary Information

Supplementary Information.

## Data Availability

Data will be made available upon request. For access, please contact Anna Omazic (anna.omazic@sva.se) or Stefan Widgren (stefan.widgren@sva.se).
