# Supplementary material for: Automated tick classification using deep learning and its associated challenges in citizen science
Source: Sci Rep. 2025 Jul 10;15:24942. doi: 10.1038/s41598-025-10265-x (PMC12246205; doi:10.1038/s41598-025-10265-x)
Supplement: Supplementary file 1 — Supplementary Information. [file 41598_2025_10265_MOESM1_ESM.pdf]

S1 Explainable AI (XAI)

Figure S1 shows additional visualizations of Explainable AI (XAI) using the RISE method for five species not shown in the main text. These supplementary saliency maps demonstrate the regions of the input images that significantly contributed to the model's predictions for the following species: *Carios vespertilionis*, *Dermacentor reticulatus*, *Haemaphysalis punctata*, *Ixodes hexagonus*, and *Ixodes ricinus*.

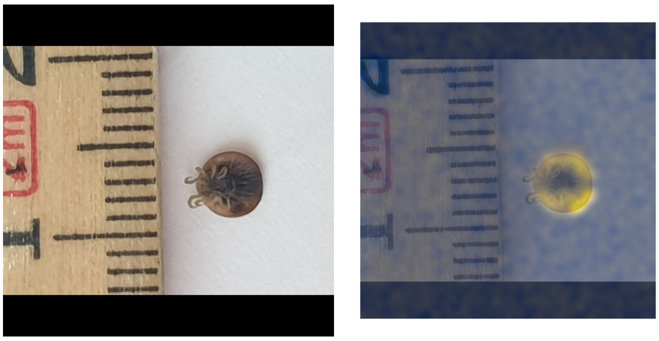

(a) Correctly classified *Carios vespertilionis*

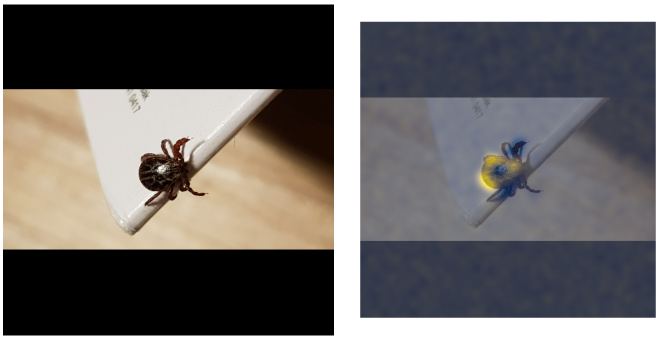

(b) Correctly classified *Dermacentor reticulatus*

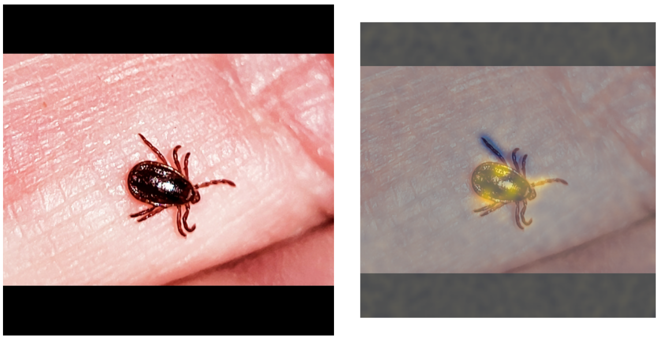

(c) Correctly classified *Haemaphysalis punctata*

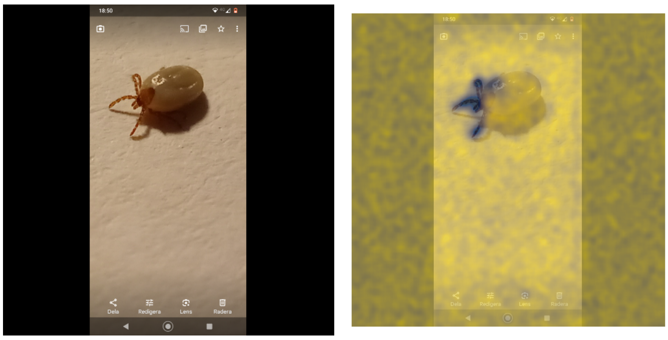

(d) Correctly classified *Ixodes hexagonus*

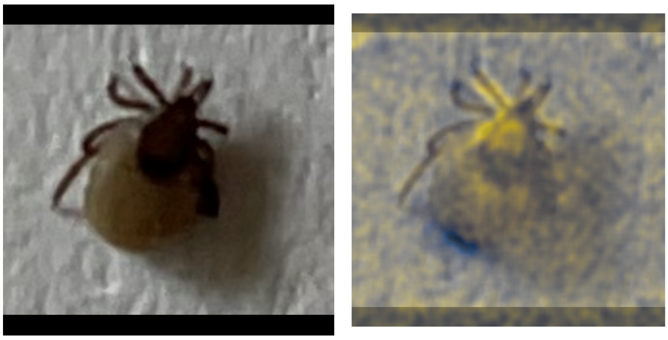

(e) Correctly classified *Ixodes ricinus*

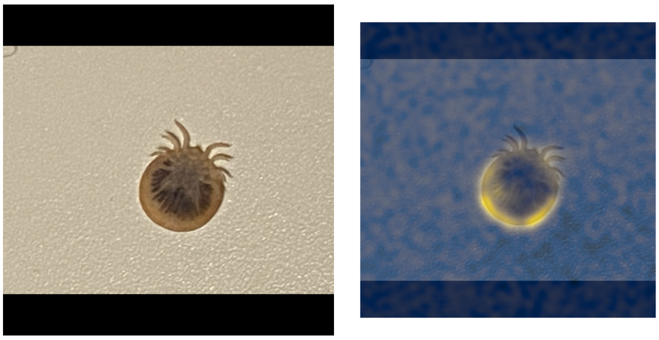

(f) Correctly classified *Carios vespertilionis*

Figure S1: Examples of saliency maps generated by the RISE method for five species not presented in the main text.

S2 Stratified Performance by Engorgement Status

To evaluate the potential impact of feeding status on model performance, we stratified image-level classification results by engorgement state (Engorged, Not Engorged, Missing) for both in-distribution (ID) and out-of-distribution (OOD) test sets. As shown in Tables S1, S2 and S3 (OOD sample level), the classifier performs consistently well on *Ixodes ricinus* regardless of engorgement, though a slight drop in recall is observed for engorged individuals in the OOD set (0.92 vs. 0.93 for non-engorged). For all other species, performance is notably poor or undefined due to small sample sizes, particularly in the ID setting, where no engorged samples are available except for *I. ricinus*. These findings suggest that the model's generalization across engorgement states is highly limited outside of dominant species, supporting concerns about morphological distortion and class imbalance. Due to the low number of annotated engorged specimens for most species, these results should be interpreted with caution.

Table S1: In-distribution (ID) image-level classification performance stratified by engorgement status.

Species	Engorgement	Precision	Recall	F1-score	Support
Carios vespertilionis	Yes	0.00	0.00	0.00	0
	No	0.00	0.00	0.00	0
	Unknown	1.00	1.00	1.00	2
Ixodes ricinus	Yes	1.00	0.98	0.99	57
	No	1.00	0.96	0.98	53
	Unknown	1.00	1.00	1.00	4

Table S2: Out-of-distribution (OOD) **image-level** classification performance stratified by engorgement status.

Species	Engorgement	Precision	Recall	F1-score	Support
Carios vespertilionis	Engorged	0.02	1.00	0.04	1
	Not Engorged	0.68	0.94	0.79	32
	Missing	0.89	1.00	0.94	8
Dermacentor reticulatus	Engorged	0.00	0.00	0.00	0
	Not Engorged	0.14	0.50	0.22	8
	Missing	0.33	1.00	0.50	1
Haemaphysalis punctata	Engorged	0.00	0.00	0.00	3
	Not Engorged	0.12	0.14	0.13	7
Hyalomma marginatum	Engorged	0.03	0.75	0.07	4
	Not Engorged	0.02	0.40	0.04	5
Ixodes hexagonus	Engorged	0.04	0.12	0.06	49
	Not Engorged	0.00	0.00	0.00	1
	Missing	0.00	0.00	0.00	2
Ixodes ricinus	Engorged	1.00	0.92	0.95	8865
	Not Engorged	1.00	0.93	0.96	6693
	Missing	1.00	0.90	0.95	198
Rhipicephalus sanguineus	Engorged	0.00	0.67	0.01	3
	Not Engorged	0.01	0.60	0.02	5

Table S3: Out-of-distribution (OOD) **sample-level** classification performance stratified by engorgement status.

Species	Engorgement	Precision	Recall	F1-score	Support
Carios vespertilionis	Engorged	0.02	1.00	0.04	1
	Not Engorged	0.59	1.00	0.74	17
	Missing	0.88	1.00	0.93	7
Dermacentor reticulatus	Engorged	0.00	0.00	0.00	0
	Not Engorged	0.17	0.67	0.27	6
	Missing	0.33	1.00	0.50	1
Haemaphysalis punctata	Engorged	0.00	0.00	0.00	1
	Not Engorged	0.12	0.14	0.13	7
Hyalomma marginatum	Engorged	0.03	1.00	0.06	2
	Not Engorged	0.03	0.67	0.06	3
Ixodes hexagonus	Engorged	0.04	0.14	0.06	36
	Not Engorged	0.00	0.00	0.00	1
	Missing	0.00	0.00	0.00	1
Ixodes ricinus	Engorged	1.00	0.92	0.96	7166
	Not Engorged	1.00	0.94	0.97	5270
	Missing	1.00	0.91	0.95	134
Rhipicephalus sanguineus	Engorged	0.00	0.50	0.01	2
	Not Engorged	0.00	0.50	0.01	2
